# Burden of illness in carbapenem-resistant *Acinetobacter baumannii* infections in US hospitals between 2014 and 2019

**DOI:** 10.1186/s12879-021-07024-4

**Published:** 2022-01-06

**Authors:** Jason M. Pogue, Yun Zhou, Hemanth Kanakamedala, Bin Cai

**Affiliations:** 1 College of Pharmacy, University of Michigan, Ann Arbor, MI USA; 2 Genesis Research Inc., Hoboken, NJ USA; 3 Shionogi Inc., 300 Campus Drive, Suite 100, Florham Park, NJ 07932 USA

**Keywords:** *Acinetobacter baumannii*, Carbapenem resistant, Mortality, ICU utilization, Readmission, Retrospective

## Abstract

**Background:**

Carbapenem-resistant (CR) *Acinetobacter baumannii* is a concerning pathogen in the USA and worldwide.

**Methods:**

To assess the comparative burden of CR vs carbapenem-susceptible (CS) *A. baumannii*, this retrospective cohort study analyzed data from adult patients in 250 US hospitals from the Premier HealthCare Database (2014–2019). The outcomes analyzed included hospital length of stay (LOS), intensive care unit (ICU) utilization, discharge status, in-hospital mortality, readmission rates and hospital charges. Logistic regression was used for univariate and multivariable assessment of the independent relationship between relevant covariates, with a focus on CR status, and in-hospital mortality.

**Results:**

2047 Patients with CR and 3476 patients with CS *A. baumannii* infections were included. CR *A. baumannii* was more commonly isolated in respiratory tract infections (CR 40.7% and CS 27.0%, *P* < 0.01), whereas CS *A. baumannii* was more frequently associated with bloodstream infections (CS 16.7% and CR 8.6%, *P* < 0.01). Patients with CR *A. baumannii* infections had higher in-hospital (CR 16.4% vs CS 10.0%; *P* < 0.01) and 30-day (CR 32.2% vs CS 21.6%; *P* < 0.01) mortality compared to those with CS infections. After adjusting for age, sex, admission source, infection site, comorbidities, and treatment with in vitro active antibiotics within 72 h, carbapenem resistance was independently associated with increased mortality (adjusted odds ratio 1.42 [95% confidence interval 1.15; 1.75], *P* < 0.01). CR infections were also associated with increases in hospital length of stay (CR 11 days vs CS 9 days; *P* < 0.01), rate of intensive care unit utilization (CR 62.3% vs CS 45.1%; *P* < 0.01), rate of readmission with *A. baumannii* infections (CR 17.8% vs CS 4.0%; *P* < 0.01) and hospital charges.

**Conclusions:**

These data suggest that the burden of illness is significantly greater for patients with CR *A. baumannii* infections and are at higher risk of mortality compared with CS infections in US hospitals.

**Supplementary Information:**

The online version contains supplementary material available at 10.1186/s12879-021-07024-4.

## Background

*Acinetobacter baumannii* has become a concerning nosocomial pathogen that can lead to serious infections including nosocomial pneumonia, bloodstream infection, skin and skin structure infection, and urinary tract infection [1, 2]. *A. baumannii* is an opportunistic, nonfermenting Gram-negative rod and its capability for long-term survival and antimicrobial resistance increases the risk of colonization and infection, particularly in the hospital setting [2, 3].

A small retrospective matched cohort study conducted in the USA in the early 2000s demonstrated that the presence of multidrug-resistant (MDR) *A. baumannii* infections in hospitalized patients was associated with increased length of stay in both the hospital and intensive care unit (ICU) compared with susceptible *A. baumannii* infections or noninfected hospitalized patients [4]. However, this study failed to demonstrate a significant impact on mortality with MDR *A. baumannii* versus non-MDR *A. baumannii* [4]*.* With the increased use of carbapenems, a large proportion of *A. baumannii* have become resistant to carbapenems in the USA and globally [1, 5–7]. Carbapenem-resistant (CR) *A. baumannii* remains an urgent threat in the USA according to the 2019 Centers for Disease Control and Prevention (CDC) antimicrobial resistance status report [8]. Previous studies have shown that CR *A. baumannii* may be associated with poor outcomes [9–12]. The mortality rates in publications over the past two decades for patients with CR, MDR, or extensively drug-resistant *A. baumannii* infections have ranged between 24 and 83% globally, and patients with multiple comorbidities have a particularly high risk of mortality due to CR *A. baumannii* infection [13, 14]. Despite these findings, contemporary data assessing the comparative burden of illness and impact on outcomes of infections due to CR versus carbapenem-susceptible (CS) infections remain limited.

The objective of the current retrospective study was to evaluate the burden of illness with regard to mortality, hospitalization, ICU utilization, discharge status, and readmission in patients infected with CR versus CS *A. baumannii* based on a large US hospital-based healthcare database over a five-year period between 2014 and 2019.

## Methods

### Study design and eligibility

This was a retrospective cohort analysis of anonymized patient data from 250 US hospitals with microbiological data for *A. baumannii* in the Premier Healthcare Database between 1st January 2014 and 30th June 2019.

**Fig. 1:**
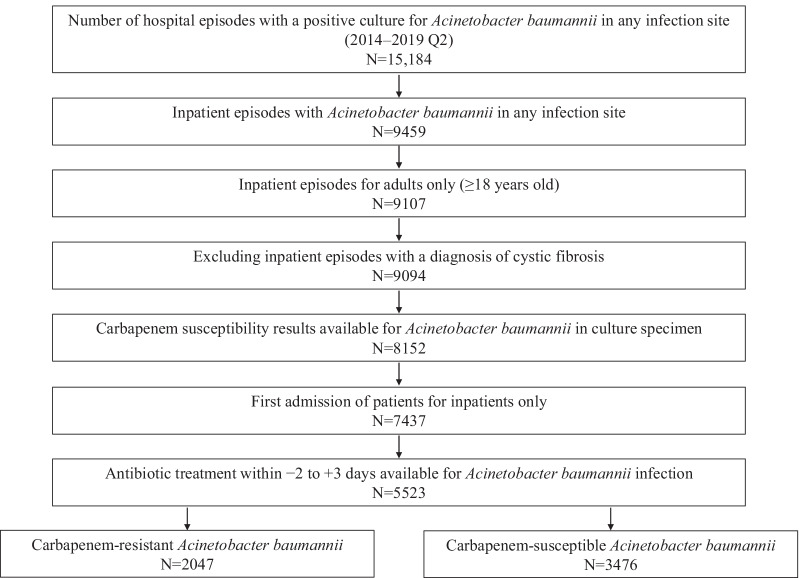
Patient attrition

Hospitalized adult patients were eligible for inclusion in the current analysis if they had isolation of *A. baumannii* at various infection sites during the study period, had microbiological susceptibility testing to the carbapenems performed, and received Gram-negative antibiotic treatment within the period of − 2 and + 3 days of the index culture to minimize the inclusion of potential colonizers (Fig. 1).

Patients were excluded from the analysis if they did not have *A. baumannii* isolated from an index culture, if they were treated in the outpatient setting, were less than 18 years of age, had cystic fibrosis, or did not have a carbapenem susceptibility test or the susceptibility test results were noninterpretable (Fig. 1).

Following Clinical and Laboratory Standards Institute breakpoints [15], the susceptibility to doripenem, imipenem, or meropenem was determined from the patient’s medical chart in order to stratify patients by carbapenem susceptibility status. *A. baumannii* was defined as carbapenem resistant (CR) if it was resistant or displayed intermediate resistance to any of these three carbapenems. *A. baumannii* was defined as carbapenem susceptible (CS) if it was susceptible to all carbapenems tested (excluding ertapenem). Because patients could have had multiple infections during the study period, the index culture and respective study group were based on the presence or absence of a CR *A. baumannii* infection occurring at any time within the study period. If patients had any CR *A. baumannii* infection during the study period, they were included in the CR cohort and the index culture was the first culture positive for CR *A. baumannii*. If patients only had CS *A. baumannii* isolated, they were included in the CS group and the index culture was the first culture positive for CS *A. baumannii* during the study period. The index hospitalization was defined as the hospitalization associated with the index culture. Each patient was only included once in the analysis.

The infection site was categorized as blood (bloodstream infection), respiratory (respiratory tract infection), urine (urinary tract infection), wound, or other. If *A. baumannii* was isolated from multiple specimens from the same patient during the index hospitalization, site of infection was defined as the site from which the first positive culture was obtained on the index culture day.

### Outcomes

The following baseline characteristics were analyzed: demographics (age [years], sex, and race), admission source (nonhealthcare or healthcare facilities), comorbidities, and site of *A. baumannii* infection (blood, respiratory, urine, wound or other). The main outcome parameters included all-cause in-hospital mortality, overall hospital length of stay (LOS) defined as the number of days from admission to discharge, and infection-associated LOS defined as the number of days from index culture date to discharge.

Other parameters included ICU admission during index hospitalization, ICU-associated LOS, infection-associated ICU utilization, infection-associated ICU-LOS, and discharge status (i.e. death, discharged to home, transferred to another institution or hospice, and other). We also assessed hospital charges related to the total duration of hospitalization, the duration of ICU stay, and infection-associated ICU stay, when applicable.

Kaplan–Meier analysis for survival probability by CR status was also performed. The rates of readmission due to any cause, with any Gram-negative organism isolated, or with repeat *A. baumannii* isolated during the readmission were analyzed for patients discharged alive from the index hospitalization.

### Statistical analysis

Descriptive statistics were assessed between patient groups, including n (%) for categorical variables and mean (standard deviation [SD]) and median (interquartile range [Q1–Q3]) for continuous variables. Univariate comparisons related to baseline characteristics and outcomes between CR and CS patients were conducted using χ^2^ test for categorical variables, a Student’s *t* test for mean values, or a Wilcoxon rank sum test for median values for continuous variables. A Kaplan–Meier analysis was conducted to analyze the days between index culture date and death date during the index hospitalization. Log-rank *P* value of < 0.05 was used for determining the statistically significant difference between CR and CS group in the probability of death at any time point.

In order to determine the independent impact of carbapenem-resistance on in-hospital mortality, crude and adjusted Odds Ratio (OR) and their 95% confidence intervals based on normal approximation (Wald) method were calculated using univariate and multivariable logistic regression. Variables included in the multivariable logistic regression were selected based on *P* value < 0.1 in the bivariate analysis of each variable and CR status.

The patient information was anonymized and deidentified prior to analysis. Because this study used already existing fully de-identified data, it was exempt from ethics review under US 45 CFR 46.104(d)(4) [16].

## Results

### Baseline characteristics

Of 15,184 screened hospital episodes with a positive culture for *A. baumannii* from any infection site during the study period, a total of 5523 patients were included in the analysis (Fig. 1). Of these, 2047 (37.1%) patients had CR and 3476 (62.9%) had CS *A. baumannii* (Table 1). Patients in the CR *A. baumannii* cohort were older (median 63 years vs 60 years; *P* < 0.01) and less likely to be male (58.9% vs 62.4%; *P* = 0.01) when compared with the CS *A. baumannii* cohort, respectively (Table 1).

**Table 1:** Baseline characteristics of patients with *Acinetobacter baumannii* infections, stratified by carbapenem susceptibility status

Characteristics	Overall *N* = 5523	Carbapenem resistant *N* = 2047	Carbapenem susceptible *N* = 3476	*P* value
Age, years, median (Q1–Q3)	61 (50.0–72.0)	63 (54.0–72.0)	60 (48.0–71.0)	< 0.01
Sex, n (%)				0.01
Female	2149 (38.9)	841 (41.1)	1308 (37.6)	
Male	3374 (61.1)	1206 (58.9)	2168 (62.4)	
Race, n (%)				< 0.01
White	3648 (66.1)	1358 (66.3)	2290 (65.9)	
Black	1155 (20.9)	500 (24.4)	655 (18.8)	
Other	641 (11.6)	151 (7.4)	490 (14.1)	
Unable to determine	79 (1.4)	38 (1.9)	41 (1.2)	
Site of index culture infection, n (%)				< 0.01
Blood	756 (13.7)	176 (8.6)	580 (16.7)	
Respiratory	1770 (32.1)	833 (40.7)	937 (27.0)	
Urine	681 (12.3)	233 (11.4)	448 (12.9)	
Wound^a^	1970 (35.7)	693 (33.9)	1277 (36.7)	
Other^b^	346 (6.3)	112 (5.5)	234 (6.7)	
Admission source, n (%)				< 0.01
Nonhealthcare facility	3936 (71.3)	1230 (60.1)	2706 (77.9)	
Transfer from other facility/hospital	1137 (20.6)	560 (27.4)	577 (16.6)	
Transfer from SNF/ intermediate care facility	345 (6.3)	237 (11.6)	108 (3.1)	
Other/unavailable	105 (1.9)	20 (1.0)	85 (2.5)	
Days between admission and index culture, n (%)				< 0.01
1 Day prior to admission	217 (3.9)	57 (2.8)	160 (4.6)	
Same day as admission	2500 (45.3)	734 (35.9)	1766 (50.8)	
Day 2	907 (16.4)	407 (19.9)	500 (14.4)	
Day 3	352 (6.4)	165 (8.1)	187 (5.4)	
Day 4	208 (3.8)	79 (3.9)	129 (3.7)	
Day 5	178 (3.2)	93 (4.5)	85 (2.5)	
≥ 6 days	1161 (21.0)	512 (25.0)	649 (18.7)	
Baseline CCI Score				
Median (Q1–Q3)	3 (2.0–5.0)	3 (2.0–5.0)	3 (1.0–5.0)	< 0.01
0, n (%)	647 (11.7)	145 (7.1)	502 (14.4)	< 0.01
1, n (%)	730 (13.2)	227 (11.1)	503 (14.5)	
2, n (%)	939 (17.0)	383 (18.7)	556 (16.0)	
3–5, n (%)	2115 (38.3)	843 (41.2)	1272 (36.6)	
5 + , n (%)	1092 (19.8)	449 (21.9)	643 (18.5)	
Baseline comorbidities, n (%)				
At least 1 comorbidity	4876 (88.3)	1902 (92.9)	2974 (85.6)	< 0.01
Myocardial infarction	681 (12.3)	266 (13.0)	415 (11.9)	0.25
Congestive heart failure	1676 (30.4)	724 (35.4)	952 (27.4)	< 0.01
Peripheral vascular disease	856 (15.5)	332 (16.2)	524 (15.1)	0.26
Cerebrovascular disease	584 (10.6)	262 (12.8)	322 (9.3)	< 0.01
Dementia	345 (6.3)	177 (8.7)	168 (4.8)	< 0.01
Chronic pulmonary disease	1798 (32.6)	780 (38.1)	1018 (29.3)	< 0.01
Peptic ulcer disease	102 (1.9)	43 (2.1)	59 (1.7)	0.28
Liver disease	514 (9.3)	157 (7.7)	357 (10.3)	< 0.01
Diabetes	2558 (46.3)	1083 (52.9)	1475 (42.4)	< 0.01
Hemiplegia or paraplegia	826 (15.0)	442 (21.6)	384 (11.1)	< 0.01
Renal disease	1849 (33.5)	772 (37.7)	1077 (31.0)	< 0.01
Any malignancy	439 (8.0)	113 (5.5)	326 (9.4)	< 0.01
Metastatic solid tumor	178 (3.2)	41 (2.0)	137 (3.9)	< 0.01
Active drug within 72 h on or after index culture date^c^	2445 (44.3)	312 (15.2)	2133 (61.4)	< 0.01

*CCI* Charlson Comorbidity Index, *SNF* Skilled nursing facility

^a^Wound, bone, body parts, skin, tissue, abscess, ulcer

^b^Abdomen, genital, sinus, other

^c^Active drug corresponded to the result of the susceptibility testing for *A. baumannii* performed on the index culture date

The median Charlson Comorbidity Index score of patients with CR and CS infections were equivalent (3 vs 3), although higher scores were more frequent in patients with CR infections (Table 1). In line with this, patients with CR infections were more likely to have any comorbid condition (92.9% vs 85.6%; *P* < 0.01) and had higher frequencies of various comorbidities, including diabetes (52.9% vs 42.4%, *P* < 0.01), chronic pulmonary disease (38.1% vs 29.3%, *P* < 0.01), renal disease (37.7% vs 31.0%, *P* < 0.01), congestive heart failure (35.4% vs 27.4%, *P* < 0.01), and hemiplegia or paraplegia (21.6% vs 11.1%, *P* < 0.01) than patients with CS *A. baumannii* infections (Table 1).

Overall *A. baumannii* was most frequently isolated from wounds (35.7%), followed by the respiratory tract (32.1%), blood (13.7%), urine (12.3%), or other sources (6.3%) (Table 1; Additional file 1: Fig. S1). The distribution of CR and CS *A. baumannii* differed significantly (*P* < 0.01) by site. Respiratory tract infections were more common with CR isolates (40.7% vs 27.0%, *P* < 0.01), while CS isolates were more common in bloodstream infections (CR 8.6% vs CS 16.7%, *P* < 0.01). CR and CS *A. baumannii* had been found in similar proportions in urine (CR 11.4% and CS 12.9%, *P* = 0.10) and wounds (CR 33.9% and CS 36.7%, *P* = 0.03) (Table 1; Additional file 1: Fig. S1).

Patients with CR infections were more likely to be transferred from another hospital/facility (27.4% vs 16.6%, *P* < 0.01), or skilled-nursing or intermediate care facility (11.6% vs 3.1%, *P* < 0.01). Approximately half of CS *A. baumannii* were detected in the index culture on the day prior to or same day of hospital admission (55.4%), while this rate was 38.6% for CR *A. baumannii* (*P* < 0.01). CR patients were more likely to have a nosocomial origin (> 3 days after hospital admission) of their pathogen (33.4% vs 24.9%; *P* < 0.01) (Table 1).

### Length of stay and ICU utilization

In CR *A. baumannii* infections, median LOS was 2 days longer (11 days vs 9 days; *P* < 0.01) and median infection-associated LOS was 1 day longer (9 days vs 8 days; *P* < 0.01) than in CS *A. baumannii* infections (Table 2). Patients with CR *A. baumannii* infections were more often admitted to the ICU for any reason (62.3% vs 45.1%; *P* < 0.01), and to have an index culture during the ICU stay (45.3% vs 33.3%; *P* < 0.01) than patients with CS *A. baumannii* infections (Table 2). There was no difference in median ICU LOS (CR 6 vs CS 6, *P* = 0.98) or infection-associated ICU LOS (CR 5 vs CS 5, *P* = 0.86) between CR and CS *A. baumannii* infections. The overall LOS charges and infection-associated LOS charges were significantly higher for CR infections than for CS infections (Additional file 2: Table S1). ICU-LOS charges and infection-associated ICU-LOS charges were similar between CR and CS *A. baumannii* infections (Additional file 2: Table S1).

**Table 2:** Outcomes in patients with *Acinetobacter baumannii* infections, stratified by carbapenem susceptibility status

Outcomes	Overall *N* = 5523	Carbapenem resistant *N* = 2047	Carbapenem susceptible *N* = 3476	*P* value
Overall LOS^a^				
Overall LOS, days, median (Q1–Q3)	10 (6.0–18.0)	11 (7.0–19.0)	9 (6.0–17.0)	< 0.01
Infection-associated LOS^b^				
Infection-associated LOS, days, median (Q1–Q3)	8 (5.0–14.0)	9 (5.0–14.0)	8 (5.0–13.0)	< 0.01
ICU utilization				
ICU admission during hospitalization, n (%)	2845 (51.5)	1276 (62.3)	1569 (45.1)	< 0.01
Index culture during ICU stay, n (%)	2084 (37.7)	927 (45.3)	1157 (33.3)	< 0.01
ICU-LOS, days, median (Q1–Q3)^c^	6 (2.0–15.0)	6 (3.0–14.0)	6 (2.0–15.0)	0.98
Infection-associated ICU utilization				
Index culture during infection-associated ICU stay, n (%)	2084 (37.7)	927 (45.3)	1157 (33.3)	< 0.01
Infection-associated ICU-LOS, days, median (Q1–Q3)^d^	5 (2.0–10.0)	5 (2.0–10.0)	5 (2.0–10.0)	0.86
Discharge status, n (%)				< 0.01
Death	683 (12.4)	336 (16.4)	347 (10.0)	
Home	1992 (36.1)	332 (16.2)	1660 (47.8)	
Hospice	236 (4.3)	98 (4.8)	138 (4.0)	
Other	92 (1.7)	14 (0.7)	78 (2.2)	
Transfer to other facility	2520 (45.6)	1267 (61.9)	1253 (36.1)	

*ICU* intensive care unit, *LOS* length of stay

^a^Defined as the number of days from admission to discharge date, regardless of discharge status

^b^Defined as the number of days from index culture date to discharge, regardless of discharge status

^c^Defined as the number of days from first to the last ICU service day, regardless of discharge status

^d^Defined as the number of days in ICU that was associated with the infection (i.e., index culture was taken within 2 days before ICU admission or during the ICU stay) and calculated as the following: (1) if a positive culture was obtained within 2 days of ICU admission, the entire ICU. LOS was used; (2) if the culture was obtained during ICU stay, the LOS was taken as the number of days from the ICU index culture to discharge from the ICU

### Mortality and discharge status

**Fig. 2:**
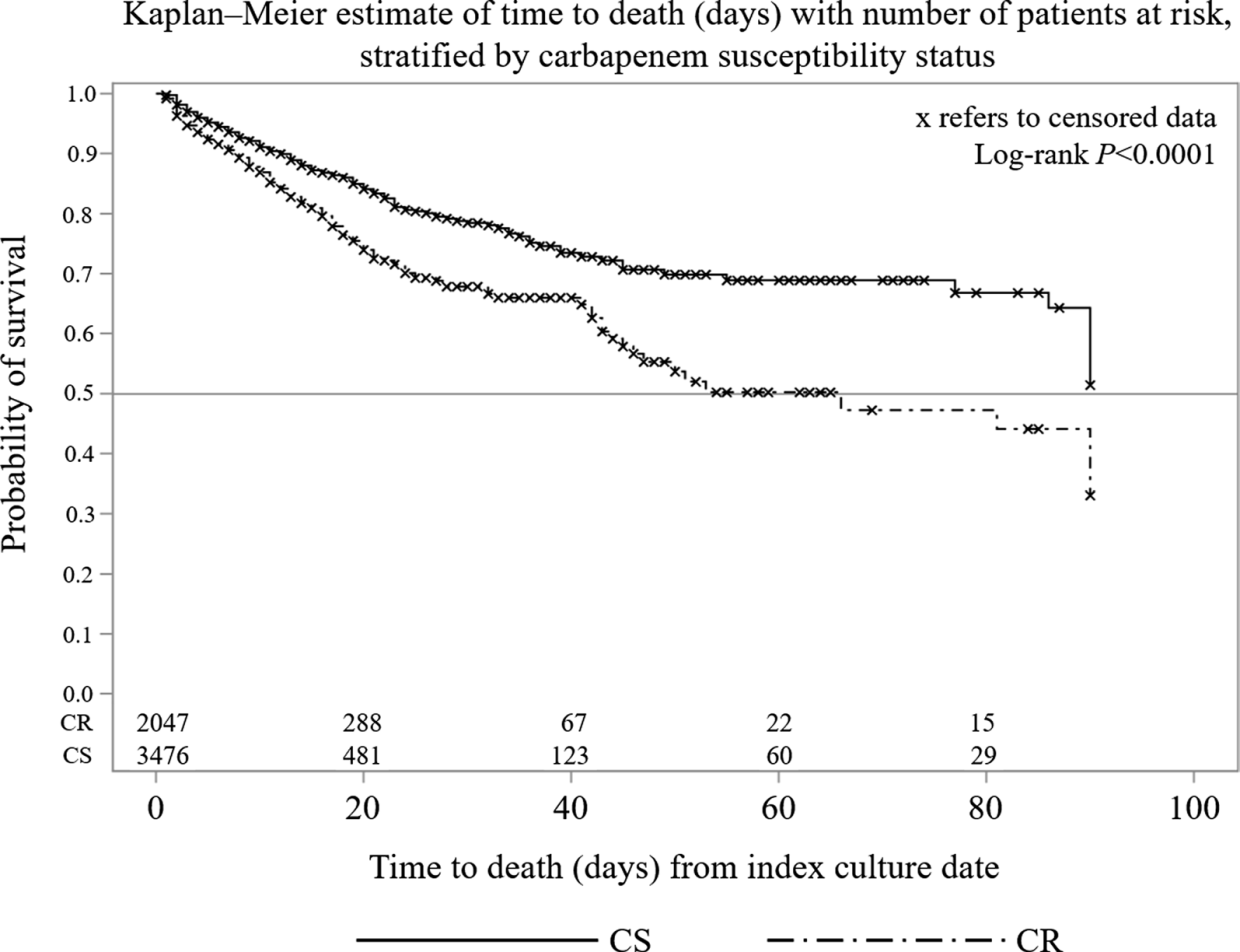
Kaplan–Meier analysis of survival probability in patients with *Acinetobacter baumannii* infections. *CR* carbapenem resistant, *CS* carbapenem susceptible

Patients with CR *A. baumannii* infections had higher in-hospital mortality (16.4% vs 10.0%; *P* < 0.01). There was also an increased mortality in patients with CR infections at Day 30 (32.2% vs 21.6%; *P* < 0.01) and Day 60 (49.8% vs 31.2%; *P* < 0.01). Based on the Kaplan–Meier analysis, patients with CR *A. baumannii* infections had a significantly higher probability of dying than those with CS *A. baumannii* infections (log-rank *P* < 0.0001) (Fig. 2). Patients with CR *A. baumannii* infections were significantly less likely to be discharged home (16.2% vs 47.8%) and significantly more likely to be discharged/transferred to another facility (61.9% vs 36.1%) than patients with CS *A. baumannii* infections (*P* < 0.01, Table 2). Patients who were admitted to hospital from a nonhealthcare facility point of origin were less likely to be discharged home if they had CR infections (19.9%) than if they had CS infections (51.3%) (*P* < 0.01).

Among patients with CR *A. baumannii* infections, the highest mortality rate was observed for bloodstream infections (40.9%; Table 3) followed by respiratory tract infections (21.9%), while mortality was 9.3% for patients with urinary tract infections and 6.6% for those with wound infections (Table 3). For patients with CS *A. baumannii* respiratory tract infections, a mortality rate of 23.2% was found, but the rate was only 12.1% for patients with bloodstream infections (Table 3). Higher mortality rates for CR than CS *A.* *baumannii* infections was observed in bloodstream, urinary tract, wound, and other types of infections, while mortality rates were similar in patients with respiratory tract infections. Overall, patients were at higher risk of mortality if they were admitted to the ICU during the index hospitalization (CR infections: 23.3% with ICU vs 5.1% without ICU; CS infections: 18.9% with ICU vs 2.6% without ICU) (Additional file 3: Table S2). The difference in mortality rates between CR and CS infections based on ICU status was consistent across infection sites (Additional file 3: Table S2).

**Table 3:** Discharge status of patients with *Acinetobacter baumannii* infections, stratified by carbapenem susceptibility status and site of infection

Discharge status, n (%)	Carbapenem-resistant *A. baumannii*						Carbapenem-susceptible *A. baumannii*					
	*N* = 2047						*N* = 3476					
	Overall *N* = 2047	Blood *N* = 176	Respiratory *N* = 833	Urine *N* = 233	Wound *N* = 693	Other *N* = 112	Overall *N* = 3476	Blood *N* = 580	Respiratory *N* = 937	Urine *N* = 448	Wound *N* = 1277	Other *N* = 234
Death	336 (16.4)	72 (40.9)	182 (21.9)	22 (9.4)	46 (6.6)	14 (12.5)	347 (10.0)	70 (12.1)	217 (23.2)	13 (2.9)	41 (3.2)	6 (2.6)
Home	332 (16.2)	21 (11.9)	85 (10.2)	49 (21.0)	153 (22.1)	24 (21.4)	1660 (47.8)	289 (49.8)	219 (23.4)	267 (59.6)	748 (58.6)	137 (58.6)
Hospice	98 (4.8)	7 (4.0)	37 (4.4)	17 (7.3)	30 (4.3)	7 (6.3)	138 (4.0)	33 (5.7)	42 (4.5)	25 (5.6)	26 (2.0)	12 (5.1)
Other	14 (0.7)	4 (2.3)	0	2 (0.9)	8 (1.2)	0	78 (2.2)	21 (3.6)	6 (0.6)	4 (0.9)	46 (3.6)	1 (0.4)
Transfer	1267 (61.9)	72 (40.9)	529 (63.5)	143 (61.4)	456 (65.8)	67 (59.8)	1253 (36.1)	167 (28.8)	453 (48.4)	139 (31.0)	416 (32.6)	78 (33.3)

Table 4 displays the association between covariates identified using bivariate analysis (Additional file 3: Table S3) and in-hospital mortality in unadjusted and adjusted regression models. Presence of CR was associated with an increased risk of death in the unadjusted analysis (unadjusted OR: 1.77; 95% confidence interval: 1.51; 2.08; *P* < 0.01). After controlling for age, race, comorbidities, site of infection, admission source, and receipt of antibiotics with in vitro activity within 72 h of index culture, CR *A. baumannii* was independently associated with an increased risk of in-hospital mortality (adjusted OR: 1.42 [1.15; 1.75], *P* < 0.01).

**Table 4:** Multivariate logistic regression analysis for in-hospital mortality with unadjusted and adjusted odds ratios and 95% confidence intervals

Risk factor	Unadjusted OR (95% CI)	*P* value	Adjusted OR (95% CI)	*P* value
Age group (reference group age 18–35)				
36–55	1.73 (1.09; 2.73)	0.02	1.67 (1.03; 2.71)	0.04
56–75	3.33 (2.16; 5.14)	< 0.01	2.45 (1.54; 3.91)	< 0.01
> 75	4.33 (2.76; 6.79)	< 0.01	3.53 (2.17; 5.74)	< 0.01
Sex				
Male vs female	0.87 (0.74; 1.02)	0.09	0.94 (0.79; 1.13)	0.51
Race (reference group “black”)				
White	1.15 (0.94; 1.42)	0.18	1.14 (0.91; 1.43)	0.27
Other	1.47 (1.11; 1.96)	0.01	1.57 (1.15; 2.15)	< 0.01
Unknown	0.79 (0.36; 1.76)	0.57	0.56 (0.24; 1.31)	0.18
Infection site (reference group “Urine”)				
Blood	4.27 (2.90; 6.28)	< 0.01	3.82 (2.56; 5.71)	< 0.01
Respiratory	5.37 (3.76; 7.68)	< 0.01	2.66 (1.80; 3.92)	< 0.01
Wound	0.85 (0.57; 1.28)	0.44	0.89 (0.58; 1.34)	0.57
Other	1.13 (0.64; 1.99)	0.67	0.99 (0.55; 1.78)	0.98
Days between admission and index culture (prior to admission)				
Same day as admission	1.29 (0.76; 2.19)	0.34	0.88 (0.5; 1.55)	0.66
2 days	1.27 (0.73; 2.21)	0.41	0.78 (0.42; 1.43)	0.42
3 days	1.75 (0.96; 3.19)	0.07	0.96 (0.50; 1.86)	0.92
4 days	2.20 (1.16; 4.16)	0.02	1.23 (0.61; 2.47)	0.56
5 days	2.45 (1.28; 4.67)	0.01	1.12 (0.55; 2.27)	0.76
≥ 6 days	3.41 (2.01; 5.79)	< 0.01	1.57 (0.87; 2.81)	0.13
CCI group (reference group CCI = 0)				
1	1.52 (0.99; 2.33)	0.06	1.10 (0.70; 1.72)	0.69
2	1.82 (1.22; 2.72)	< 0.01	1.37 (0.90; 2.11)	0.14
3–5	2.54 (1.77; 3.63)	< 0.01	1.72 (1.17; 2.54)	0.01
> 5	4.31 (2.98; 6.21)	< 0.01	2.73; (1.83; 4.08)	< 0.01
Admission source (reference group “Home”)				
Nursing	1.72 (1.29; 2.31)	< 0.01	1.22 (0.88; 1.68)	0.23
Transferred from other facilities	1.39 (1.15; 1.68)	< 0.01	1.17 (0.95; 1.45)	0.14
Other	0.48 (0.21; 1.09)	0.08	0.63 (0.26; 1.51)	0.30
Infection associated ICU utilization				
Yes vs no	4.77 (4.01; 5.68)	< 0.01	2.93 (2.40; 3.57)	< 0.01
Carbapenem susceptibility status				
CR vs CS	1.77 (1.51; 2.08)	< 0.01	1.42 (1.15; 1.75)	< 0.01
Patient received any active drug within 72 h of index culture date				
No active antibiotic^a^ vs active antibiotic^b^	1.16 (0.98; 1.36)	0.08	1.03 (0.84; 1.27)	0.77

*CCI* Charlson Comorbidity Index, *CI* Confidence interval, *CR* Carbapenem resistant, *CS* Carbapenem susceptible, *ICU* intensive care unit, *OR* odds ratio

^a^The antibiotic is considered as “active” if *Acinetobacter baumannii* was “susceptible” based on susceptibility testing result. The category included tigecycline and colistin without available susceptibility testing result

^b^The antibiotic is considered as “not active” if *Acinetobacter baumannii* was “resistant” or “intermediate” based on susceptibility testing result or if antibiotics were not tested for susceptibility or the missing testing results cannot be imputed based on the algorithm in Additional file 7: Table S6

### Readmission

There was no difference in rates of overall readmission due to any cause between patients in the CR and CS *A. baumannii* cohorts (CR 58.4% vs CS 56.8%) who were alive at discharge. Compared with patients with CS *A. baumannii* infections, the readmission with any Gram-negative organism isolated (CR 36.4% vs CS 22.2%; *P* < 0.01) and with *A. baumannii* isolated (CR 17.8% vs CS 4.0%; *P* < 0.01) were markedly higher for patients with CR infections (Additional file 5: Table S4, Additional file 6: Table S5). The difference in readmission rates between patients in the CR and CS *A. baumannii* cohorts was consistent across all infection sites (Additional file 6: Table S5).

## Discussion

The current analysis provides information on the burden of illness due to CR versus CS *A. baumannii* infections among over 5000 hospitalized patients across 250 hospitals with microbiology data based on the Premier HealthCare Database between 2014 and 2019. Outcomes in patients with CR *A. baumannii* infections were worse than in those with CS infections. Patients with CR infections had an increased in-hospital mortality, increased length of overall hospital and infection-associated stay, a lower proportion of survivors being discharged home, and more frequent readmissions with isolation of the same pathogen. Increased mortality with CR infections persisted even after adjusting for other factors. Interestingly, despite more frequent ICU admission during index hospitalization, the length of ICU stay and ICU-related hospital charges were similar between CR and CS *A. baumannii* infections.

Overall, the absolute difference in in-hospital mortality rates between CR and CS infections was approximately 6% (CR 16% vs CS 10%). However, the Kaplan–Meier analysis suggested that long-term probability of survival among patients with CR infections decreased at a faster rate than among those with CS infections. We also found that the 30-day and 60-day mortality rates were also increased for patients with CR *A. baumannii* infections (CR 32.2% vs CS 21.6% at Day 30, and CR 49.8% vs CS 31.2% at Day 60). Several studies have shown a higher risk of mortality among patients with CR *A. baumannii* infections, particularly for infections occurring in the bloodstream and respiratory tract [13], with rates approaching ~ 50–60% globally. In the current study, the highest mortality rate in CR *A. baumannii* was found in bloodstream infections (> 40%), followed by respiratory tract infections (22%).

The most frequent infection sites among CR infections were the respiratory tract (> 40%) and wounds (> 30%). Most patients in this study had at least one comorbidity. The most frequently observed conditions were diabetes, chronic respiratory disease, renal disease, and congestive heart failure. Approximately 40% of CR infections and 20% of CS infections were admitted from a healthcare facility/origin. While the majority of infections were acquired in the first 48 h of hospitalization in both groups (58.6% for CR vs 69.8% for CS), there was a clear trend of later onset for CR isolates (*P* < 0.01), suggesting that CR infections were more likely than CS infections to be nosocomial in origin. Despite improved prevention efforts in US healthcare settings and hospitals, a high rate of hospital-acquired CR *A. baumannii* infections has also recently been shown in a large patient cohort [17].

A large proportion of patients with CR infections had a stay in ICU and had their index culture taken during ICU stay more frequently than those with CS infections. This suggests a high risk of acquiring CR *A. baumannii* during an ICU stay, which is consistent with previous reports [7]. This is an important finding as there was increased mortality when patients had an ICU stay during index hospitalization, which was observed among both CR and CS infections. A previous US study assessing patients in the ICU with bloodstream infections due to *Acinetobacter* spp. had suggested that inappropriate antibiotic use, particularly for CR *Acinetobacter* spp., was strongly associated with increased mortality risk [18]. Interestingly, in this analysis, no association between receipt of in vitro active antibiotics within the first 72 h after culture collection and mortality was demonstrated, even though this occurred with four times greater frequency (61% vs 15%) than in patients with CS isolates. While this analysis was not intended to address the impact of active therapy on outcomes, potential reasons for this unexpected finding include the inclusion of patients presenting with a wide degree of initial severity of illness, the large proportion of patients with less severe infection types (i.e., wound and urine), or the inferior definitive therapy options available for the treatment of carbapenem-resistant *A. baumannii*, given the extensively drug-resistant nature of most isolates.

Compared with CS patients, CR patients incurred significantly higher charges for overall LOS and infection-associated LOS, but similar charges for ICU-LOS and infection associated ICU-LOS. This may indicate that infections increased the overall hospital burden. The increased LOS and infection-associated LOS may explain the higher amount of charges for CR infections in general. Because ICU-associated LOS was not significantly different between CR and CS infections, it may be the explanation for similar charges for ICU stay by hospitals or higher mortality in CR patients resulted in a short ICU-LOS.

Readmission among patients surviving the index hospitalization may be linked to infectious or noninfectious causes because comorbidities were common in all patients. The overall readmission rates were similar between CR and CS infections. However, CR patients were more likely to be readmitted and have isolation of a Gram-negative organism, or with *A. baumannii* specifically, which may suggest suboptimal antibiotic treatment during the index hospitalization and/or persistence of pathogens. Further investigation into optimal treatment strategies for *A. baumannii* are clearly warranted.

One limitation of the current analysis is that specific antibiotic regimens were not evaluated and therefore an association between different regimens and patient outcomes was not assessed. Further analyses are required to determine which antibiotic regimens are optimal for the treatment of *A. baumannii* infections and whether agent selection can positively impact mortality, LOS, and/or readmission rates. An additional limitation is that infections were defined in this analysis by the type of culture sample and the administration of antibiotics at the time of that index culture. While the prescription of antibiotics during the 48–72-h window surrounding the index culture is highly suggestive of infection, whether there was an infection at the culture site and/or whether *A. baumannii* was considered the primary pathogen cannot be ascertained from such an analysis.

## Conclusions

Patients with CR *A. baumannii* infections had an increased disease burden and higher rate of nosocomial infections, increased LOS and infection-associated LOS, more frequent ICU admission, and higher readmission rates than patients with CS infections. Mortality was also significantly higher for CR *A. baumannii* infections, particularly among those with bloodstream infections.

## Supplementary Information

**Additional file 1: Fig. S1.** Distribution of site of carbapenem resistant (CR) or carbapenem susceptible (CS) *Acinetobacter baumannii* infections in hospitalized patients.

**Additional file 2: Table S1.** Charges associated with length of stay and intensive care unit utilization

**Additional file 3: Table S2.** Discharge status of patients with *Acinetobacter baumannii* infections, stratified by carbapenem susceptibility status, infection site, and intensive care unit stay.

**Additional file 4: Table S3.** Univariate analysis for patient characteristics, infection-associated ICU utilization, carbapenem susceptibility status, and administration of active or inactive antibiotics for in-hospital mortality.

**Additional file 5: Table S4.** Readmission rates of patients with *Acinetobacter baumannii* infections for survivors of index hospitalization, stratified by carbapenem susceptibility status, discharge location, and infectious agent.

**Additional file 6: Table S5.** Readmission rates of patients with *Acinetobacter baumannii* infections for survivors of index hospitalization, stratified by carbapenem susceptibility status, site of infection, and infectious agent.

**Additional file 7: Table S6.** Algorithm to impute antibiotic susceptibility for *Acinetobacter baumannii* without susceptibility testing result for treatment given within 72 h of index culture.

## Data Availability

This study is conducted using an anonymous, commercially available secondary healthcare database called Premier Healthcare Database that meets the US HIPPA requirement. The data is not sharable per our license agreement with the data owner.

## Abbreviations

CCI: Charlson comorbidity index
CDC: Centers for Disease Control and Prevention
CI: Confidence interval
CR: Carbapenem resistant
CS: Carbapenem susceptible
ICU: Intensive care unit
LOS: Length of stay
MDR: Multidrug resistant
OR: Odds ratio
SD: Standard deviation
SNF: Skilled nursing facility
US: United States
