# Supplementary material for: Burden of illness in carbapenem-resistant Acinetobacter baumannii infections in US hospitals between 2014 and 2019
Source: BMC Infect Dis. 2022 Jan 6;22:36. doi: 10.1186/s12879-021-07024-4 (PMC8740340; doi:10.1186/s12879-021-07024-4)

**Figure S1.** Distribution of site of carbapenem resistant (CR) or carbapenem susceptible (CS) *Acinetobacter baumannii* infections in hospitalized patients


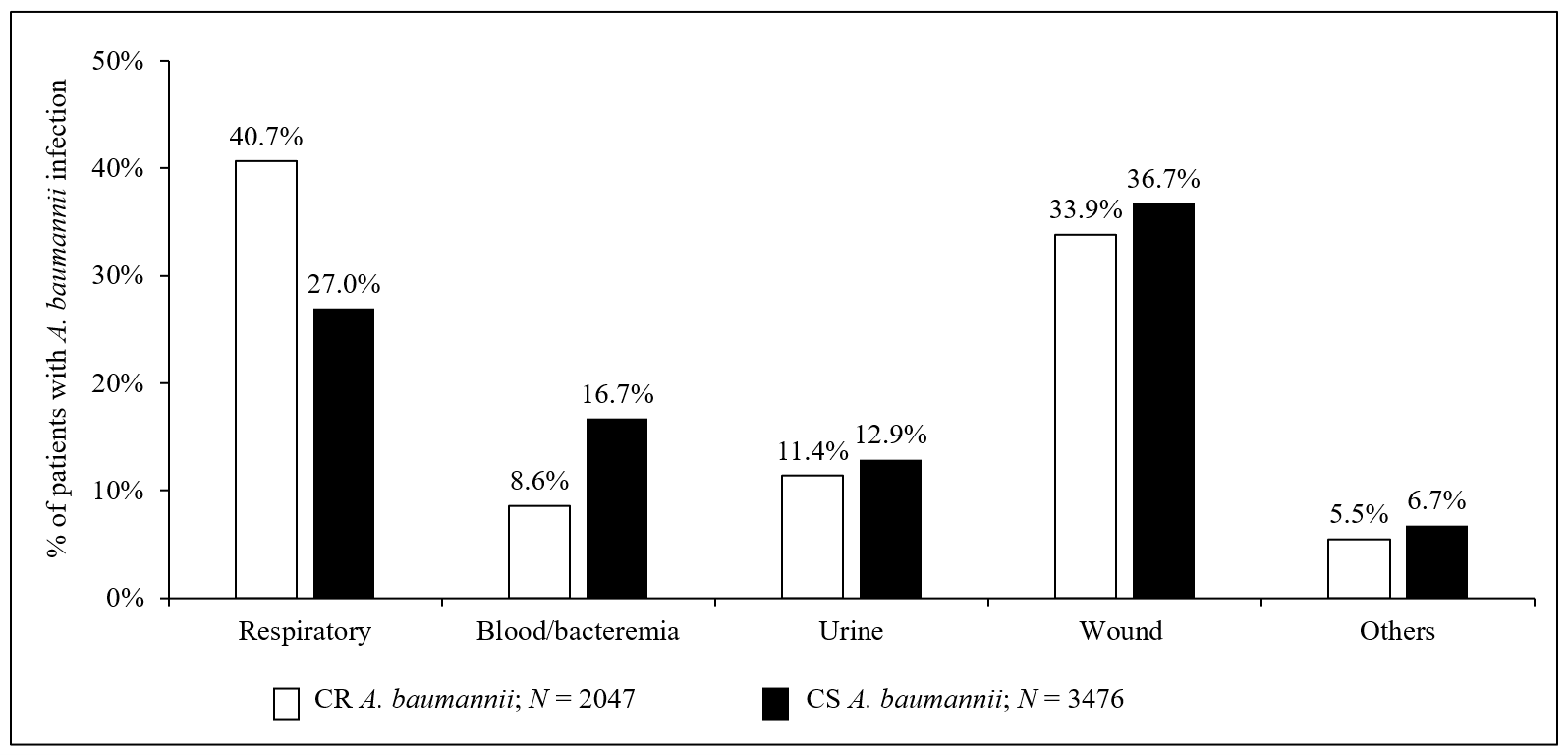

Supplement: Supplementary file 1 — Additional file 1: Fig. S1. Distribution of site of carbapenem resistant (CR) or carbapenem susceptible (CS) Acinetobacter baumannii infections in hospitalized patients. [file 12879_2021_7024_MOESM1_ESM.docx]
