# Supplementary material for: Burden of illness in carbapenem-resistant Acinetobacter baumannii infections in US hospitals between 2014 and 2019
Source: BMC Infect Dis. 2022 Jan 6;22:36. doi: 10.1186/s12879-021-07024-4 (PMC8740340; doi:10.1186/s12879-021-07024-4)
Supplement: Supplementary file 2 — Additional file 2: Table S1. Charges associated with length of stay and intensive care unit utilization [file 12879_2021_7024_MOESM2_ESM.docx]

**Table S1.** Charges associated with length of stay and intensive care unit utilization

| Charges, US$, median (Q1–Q3) | Overall  *N* = 5523 | Carbapenem resistant  *N* = 2047 | Carbapenem susceptible  *N* = 3476 | *P* value |
| --- | --- | --- | --- | --- |
| Total overall charges | 77,679 (39,723.4–167,346.2) | 97,959 (54,233.2–193,468.8) | 65,449 (34,422.6–151,964.2) | <0.01 |
| Total infection-associated charges | 58,702 (30,918.2–117,060.1) | 70,924 (37,437.4–133,459.7) | 52,254 (28,172.1–105,781.6) | <0.01 |
| Total ICU charges | 20,900 (7222.3–54,382.3) | 21,276 (8550.0–48,923.2) | 20,704 (6529.6–58,879.3) | 1.00 |
| Total infection-associated ICU charges | 17,709 (7010.9–39,177.6) | 17,130 (7364.3–37,504.0) | 18,244 (6720.0–40,286.4) | 0.79 |

*ICU*, intensive care unit.
