# Supplementary material for: Burden of illness in carbapenem-resistant Acinetobacter baumannii infections in US hospitals between 2014 and 2019
Source: BMC Infect Dis. 2022 Jan 6;22:36. doi: 10.1186/s12879-021-07024-4 (PMC8740340; doi:10.1186/s12879-021-07024-4)
Supplement: Supplementary file 3 — Additional file 3: Table S2. Discharge status of patients with Acinetobacter baumannii infections, stratified by carbapenem susceptibility status, infection site, and intensive care unit stay. [file 12879_2021_7024_MOESM3_ESM.docx]

**Table S2.** Discharge status of patients with *Acinetobacter baumannii* infections, stratified by carbapenem susceptibility status, infection site, and intensive care unit stay

| Overall *N* = 5523 | | | | | | | | | | | | | | | | | |  |
| --- | --- | --- | --- | --- | --- | --- | --- | --- | --- | --- | --- | --- | --- | --- | --- | --- | --- | --- |
| Patients without ICU stay during hospitalization *N* = 2678 | | | | | | | | | | | | | | | | | |  |
|  | Carbapenem resistant *N* = 771 | | | | | | Carbapenem susceptible *N* = 1097 | | | | | | | | | | |  |
| Discharge status, n (%) | Overall  *N* = 771 | Blood  *N* = 53 | Respiratory  *N* = 155 | Urine  *N* = 126 | Wound  *N* = 385 | Other  *N* = 52 | Overall  *N* = 1097 | Blood  *N* = 315 | | Respiratory  *N* = 184 | | Urine  *N* = 304 | | Wound  *N* = 948 | | Other  *N* = 156 | |  |
| Death | 39 (5.1) | 11 (20.8) | 13 (8.4) | 7 (5.6) | 7 (1.8) | 1 (1.9) | 50 (2.6) | 17 (5.4) | | 18 (9.8) | | 2 (0.7) | | 11 (1.2) | | 2 (1.3) | |  |
| Home | 196 (25.4) | 11 (20.8) | 25 (16.1) | 35 (27.8) | 108 (28.1) | 17 (32.7) | 1193 (62.6) | 187 (59.4) | | 81 (44.0) | | 196 (64.5) | | 619 (65.3) | | 110 (70.5) | |  |
| Hospice | 28 (3.6) | 3 (5.7) | 6 (3.9) | 5 (4.0) | 12 (3.1) | 2 (3.9) | 60 (3.2) | 15 (4.8) | | 8 (4.4) | | 14 (4.6) | | 18 (1.9) | | 5 (3.2) | |  |
| Other | 8 (1.0) | 3 (5.7) | 0 | 1 (0.8) | 4 (1.0) | 0 | 59 (3.1) | 15 (4.8) | | 2 (1.1) | | 4 (1.3) | | 37 (3.9) | | 1 (0.6) | |  |
| Transfer | 500 (64.9) | 25 (47.2) | 111 (71.6) | 78 (61.9) | 254 (66.0) | 32 (61.5) | 545 (28.6) | 81 (25.7) | | 75 (40.8) | | 88 (29.0) | | 263 (27.7) | | 38 (24.4) | |  |
| Patients with ICU stay during hospitalization *N* = 2845 | | | | | | | | | | | | | | | | | |  |
|  | Carbapenem resistant *N* = 1276 | | | | | | Carbapenem susceptible *N* = 1569 | | | | | | | | | | |  |
| Discharge status, n (%) | Overall  *N* = 1276 | Blood  *N* = 123 | Respiratory  *N* = 678 | Urine  *N* = 107 | Wound  *N* = 308 | Other  *N* = 60 | Overall  *N* = 1569 | Blood  *N* = 265 | | Respiratory  *N* = 753 | | Urine  *N* = 144 | | Wound  *N* = 329 | | Other  *N* = 78 | |  |
| Death | 297 (23.3) | 61 (49.6) | 169 (24.9) | 15 (14.0) | 39 (12.7) | 13 (21.7) | 297 (18.9) | 53 (20.0) | | 199 (26.4) | | 11 (7.6) | | 30 (9.1) | | 4 (5.1) | |  |
| Home | 136 (10.7) | 10 (8.1) | 60 (8.9) | 14 (13.1) | 45 (14.6) | 7 (11.7) | 467 (29.8) | 102 (38.5) | | 138 (18.3) | | 71 (49.3) | | 129 (39.2) | | 27 (34.6) | |  |
| Hospice | 70 (5.5) | 4 (3.3) | 31 (4.6) | 12 (11.2) | 18 (5.8) | 5 (8.3) | 78 (5.0) | 18 (6.8) | | 34 (4.5) | | 11 (7.6) | | 8 (2.4) | | 7 (9.0) | |  |
| Other | 6 (0.5) | 1 (0.8) | 0 | 1 (0.9) | 4 (1.3) | 0 | 19 (1.2) | 6 (2.3) | | 4 (0.5) | | 0 | | 9 (2.7) | | 0 | |  |
| Transfer | 767 (60.1) | 47 (38.2) | 418 (61.7) | 65 (60.8) | 202 (65.6) | 35 (58.3) | 708 (45.1) | 86 (32.5) | | 378 (50.2) | | 51 (35.4) | | 153 (46.5) | | 40 (51.3) | |  |
| Patients with infection-associated ICU during hospital stay *N* = 2357. | | | | | | | | | | | | | | | | | |  |
|  | Carbapenem resistant *N* = 1044 | | | | | | Carbapenem susceptible *N* = 1313 | | | | | | | | | | |  |
| Discharge status, n (%) | Overall  *N* = 1044 | Blood  *N* = 101 | Respiratory  *N* = 595 | Urine  *N* = 86 | Wound  *N* = 219 | Other  *N* = 43 | Overall  *N* = 1313 | | Blood  *N* = 226 | | Respiratory  *N* = 684 | | Urine  *N* = 105 | | Wound  *N* = 232 | | Other  *N* = 66 | |
| Death | 253 (24.2) | 53 (52.5) | 147 (24.7) | 10 (11.6) | 31 (14.2) | 12 (27.9) | 273 (20.8) | 50 (22.1) | | 187 (27.3) | | 9 (8.6) | | 23 (9.9) | | 4 (6.1) | |  |
| Home | 105 (10.1) | 8 (7.9) | 51 (8.6) | 10 (11.6) | 31 (14.2) | 5 (11.6) | 361 (27.5) | 84 (37.2) | | 118 (17.3) | | 52 (49.5) | | 84 (36.2) | | 23 (34.9) | |  |
| Hospice | 60 (5.8) | 4 (4.0) | 29 (4.9) | 9 (10.5) | 13 (5.9) | 5 (11.6) | 69 (5.3) | 17 (7.5) | | 32 (4.7) | | 8 (7.6) | | 6 (2.6) | | 6 (9.1) | |  |
| Other | 5 (0.5) | 0 | 0 | 1 (1.2) | 4 (1.8) | 0 | 13 (1.0) | 4 (1.8) | | 4 (0.6) | | 0 | | 5 (2.2) | | 0 | |  |
| Transfer | 621 (59.5) | 36 (35.6) | 368 (61.9) | 56 (65.1) | 140 (63.9) | 21 (48.8) | 597 (45.5) | 71 (31.4) | | 343 (50.2) | | 36 (34.3) | | 114 (49.1) | | 33 (50.0) | |  |

*ICU*, intensive care unit.
