# Supplementary material for: Burden of illness in carbapenem-resistant Acinetobacter baumannii infections in US hospitals between 2014 and 2019
Source: BMC Infect Dis. 2022 Jan 6;22:36. doi: 10.1186/s12879-021-07024-4 (PMC8740340; doi:10.1186/s12879-021-07024-4)
Supplement: Supplementary file 4 — Additional file 4: Table S3. Univariate analysis for patient characteristics, infection-associated ICU utilization, carbapenem susceptibility status, and administration of active or inactive antibiotics for in-hospital mortality. [file 12879_2021_7024_MOESM4_ESM.docx]

**Table S3.** Univariate analysis for patient characteristics, infection-associated ICU utilization, carbapenem susceptibility status, and administration of active or inactive antibiotics for in-hospital mortality

| Characteristics | Overall  *N* = 5523 | Died *N* = 683 | Survived *N* = 4840 | *P* value |
| --- | --- | --- | --- | --- |
| Age, years, n (%) |  |  |  | <0.01 |
| 18–35 | 483 (8.8) | 23 (3.4) | 460 (9.5) |  |
| 36–55 | 1496 (27.1) | 119 (17.4) | 1377 (28.5) |  |
| 56–75 | 2573 (46.6) | 368 (53.9) | 2205 (45.6) |  |
| >75 | 971 (17.6) | 173 (25.3) | 798 (16.5) |  |
| Sex, n (%) |  |  |  | 0.09 |
| Female | 2149 (38.9) | 286 (41.9) | 1863 (38.5) |  |
| Male | 3374 (61.1) | 397 (58.1) | 2977 (61.5) |  |
| Race, n (%) |  |  |  | 0.04 |
| White | 3648 (66.1) | 452 (66.2) | 3196 (66.0) |  |
| Black | 1155 (20.9) | 126 (18.5) | 1029 (21.3) |  |
| Other | 641 (11.6) | 98 (14.4) | 543 (11.2) |  |
| Unable to determine | 79 (1.4) | 7 (1.0) | 72 (1.5) |  |
| Site of index culture infection, n (%) <0.01 | | | | |
| Blood | 756 (13.7) | 142 (20.8) | 614 (12.7) |  |
| Respiratory | 1770 (32.1) | 399 (58.4) | 1371 (28.3) |  |
| Urine | 681 (12.3) | 35 (5.1) | 646 (13.4) |  |
| Wound^a^ | 1970 (35.7) | 87 (12.7) | 1883 (38.9) |  |
| Other^b^ | 346 (6.3) | 20 (2.9) | 326 (6.7) |  |
| Admission source, n (%) <0.01 | | | | |
| Nonhealthcare facility | 3936 (71.3) | 444 (65.0) | 3492 (72.2) |  |
| Transfer from other facility/hospital | 1137 (20.6) | 171 (25.0) | 966 (20.0) |  |
| Transfer from SNF/ intermediate care facility | 345 (6.3) | 62 (9.1) | 283 (5.9) |  |
| Other/unavailable | 105 (1.9) | 6 (0.9) | 99 (2.1) |  |
| Days between admission and index culture, n (%) <0.01 | | | | |
| 1 day prior to admission | 217 (3.9) | 16 (2.3) | 201 (4.2) |  |
| Same day as admission | 2500 (45.3) | 233 (34.1) | 2267 (46.8) |  |
| Day 2 | 907 (16.4) | 83 (12.2) | 824 (17.0) |  |
| Day 3 | 352 (6.4) | 43 (6.3) | 309 (6.4) |  |
| Day 4 | 208 (3.8) | 31 (4.5) | 177 (3.7) |  |
| Day 5 | 178 (3.2) | 29 (4.3) | 149 (3.1) |  |
| ≥6 days | 1161 (21.0) | 248 (36.3) | 913 (18.9) |  |
| Baseline CCI Score, n (%) <0.01 | | | | |
| 0 | 647 (11.7) | 36 (5.3) | 611 (12.6) |  |
| 1 | 730 (13.2) | 60 (8.8) | 670 (13.8) |  |
| 2 | 939 (17.0) | 91 (13.3) | 848 (17.5) |  |
| 3–5 | 2115 (38.3) | 275 (40.3) | 1840 (38.0) |  |
| 5+ | 1092 (19.8) | 221 (32.4) | 871 (18.0) |  |
| Infection-associated ICU, n (%) <0.01 | | | | |
| Yes | 2084 (37.7) | 480 (70.3) | 1604 (33.1) |  |
| No | 3439 (62.3) | 203 (29.7) | 3,36 (66.9) |  |
| Carbapenem susceptibility, n (%) | |  |  | <0.01 |
| CR | 2047 (37.1) | 336 (49.2) | 1711 (35.4) |  |
| CS | 3476 (62.9) | 347 (50.8) | 3129 (64.6) |  |
| Active drug within 72 hours on or after index culture date, n (%) | | | | 0.08 |
| Active^a^ | 2445 (44.3) | 281 (41.1) | 2164 (44.7) |  |
| Inactive^b^ | 3078 (55.7) | 402 (58.9) | 2676 (55.3) |  |

*CCI* Charlson Comorbidity Index; *CR* Carbapenem resistant; *CS* Carbapenem susceptible, *ICU* intensive care unit; *SNF* skilled nurse facility.

^a^ The antibiotic is considered as “active” if *Acinetobacter baumannii* was “susceptible” based on susceptibility testing result. The category included tigecycline and colistin without available susceptibility testing result.

^b^ The antibiotic is considered as "not active" if *Acinetobacter baumannii* was “resistant” or “intermediate" based on susceptibility testing result or if antibiotics were not tested for susceptibility or the missing testing results cannot be imputed based on the algorithm in Additional File 7, Supplementary Table 6.
