# Supplementary material for: Burden of illness in carbapenem-resistant Acinetobacter baumannii infections in US hospitals between 2014 and 2019
Source: BMC Infect Dis. 2022 Jan 6;22:36. doi: 10.1186/s12879-021-07024-4 (PMC8740340; doi:10.1186/s12879-021-07024-4)
Supplement: Supplementary file 5 — Additional file 5: Table S4. Readmission rates of patients with Acinetobacter baumannii infections for survivors of index hospitalization, stratified by carbapenem susceptibility status, discharge location, and infectious agent. [file 12879_2021_7024_MOESM5_ESM.docx]

**Table S4.** Readmission rates of patients with *Acinetobacter baumannii* infections for survivors of index hospitalization, stratified by carbapenem susceptibility status, discharge location, and infectious agent

| Readmission | Carbapenem resistant *N* = 1711 | | | | | Carbapenem susceptible *N* = 3129 | | | | |
| --- | --- | --- | --- | --- | --- | --- | --- | --- | --- | --- |
|  | Overall  *N* = 1711 | Discharged to hospice  *N* = 98 | Discharged to home  *N* = 332 | Transferred to other facility  *N* = 1267 | Other  *N* = 14 | Overall  *N* = 3129 | Discharged to hospice  *N* = 138 | Discharged to home  *N* = 1660 | Transferred to other facility  *N* = 1253 | Other  *N* = 78 |
| Readmission due to any cause, n (%)^*^ | | | | | | | | | | |
| Overall | 999 (58.4) | 24 (24.5) | 237 (71.4) | 729 (57.5) | 9 (64.3) | 1778 (56.8) | 30 (21.7) | 1028 (61.9) | 671 (53.6) | 49 (62.8) |
| Within 30 days | 432 (25.3) | 22 (22.5) | 105 (31.6) | 299 (23.6) | 6 (42.9) | 703 (22.5) | 27 (19.6) | 386 (23.3) | 265 (21.2) | 25 (32.1) |
| Days between discharge from index hospitalization to first readmission, median (Q1–Q3) | 41 (12.0–105.0) | 0 (0.0–2.0) | 40 (14.0–116.0) | 44 (13.0–106.0) | 17 (3.0–161.0) | 51 (13.0–163.0) | 0 (0.0–0.0) | 56 (16.0–174.50) | 52 (11.0–161.0) | 26 (12.0–146.0) |
| Readmission with any Gram-negative organism isolated, n (%)^*,†^ | | | | | | | | | | |
| Overall | 623 (36.4) | 5 (5.1) | 145 (43.7) | 468 (36.9) | 5 (35.7) | 694 (22.2) | 4 (2.9) | 393 (23.7) | 281 (22.4) | 16 (20.5) |
| Within 30 days | 197 (11.5) | 4 (4.1) | 45 (13.6) | 145 (11.4) | 3 (21.4) | 176 (5.6) | 1 (0.7) | 95 (5.7) | 71 (5.7) | 9 (11.5) |
| Days between discharge from index hospitalization to first readmission, median (Q1–Q3) | 58 (23.0–151.0) | 12 (5.0–24.0) | 56 (23.0–156.0) | 62 (23.0–151.5) | 27 (20.0–34.0) | 94 (30.0–246.0) | 62.5 (43.5–677.5) | 96 (31.0–261.0) | 99 (30.0–227.0) | 24 (15.0–121.0) |
| Readmission with *A. baumannii*, n (%)^*^ | | | | | | | | | | |
| Overall | 305 (17.8) | 4 (4.1) | 71 (21.4) | 228 (18.0) | 2 (14.3) | 125 (4.0) | 0 | 72 (4.3) | 48 (3.8) | 5 (6.4) |
| Within 30 days | 106 (6.2) | 4 (4.1) | 23 (6.9) | 78 (6.2) | 1 (7.1) | 41 (1.3) | 0 | 22 (1.3) | 15 (1.2) | 4 (5.1) |
| Days between discharge from index hospitalization to first readmission, median (Q1–Q3) | 51 (20.0–122.0) | 8.5 (4.0–18.0) | 49 (20.0–137.0) | 55 (21.5–122.5) | 40.5 (3.0–78.0) | 79 (18.0–185.0) | – | 97 (23.0–227.0) | 68 (19.5–155.0) | 17 (12.0–20.0) |

^*^Unless otherwise specified.

^†^Most commonly isolated Gram-negative organisms included *Escherichia coli*, *Enterobacter cloacae*, *Klebsiella aerogenes*, *Klebsiella oxytoca*, *Klebsiella pneumoniae*, *Morganella morganii*, *Proteus mirabilis*, *Serratia marcescens*, *Acinetobacter baumannii*, *Pseudomonas aeruginosa*, *Stenotrophomonas maltophilia*.
