# Supplementary material for: Burden of illness in carbapenem-resistant Acinetobacter baumannii infections in US hospitals between 2014 and 2019
Source: BMC Infect Dis. 2022 Jan 6;22:36. doi: 10.1186/s12879-021-07024-4 (PMC8740340; doi:10.1186/s12879-021-07024-4)
Supplement: Supplementary file 6 — Additional file 6: Table S5. Readmission rates of patients with Acinetobacter baumannii infections for survivors of index hospitalization, stratified by carbapenem susceptibility status, site of infection, and infectious agent. [file 12879_2021_7024_MOESM6_ESM.docx]

**Table S5.** Readmission rates of patients with *Acinetobacter baumannii* infections for survivors of index hospitalization, stratified by carbapenem susceptibility status, site of infection, and infectious agent

| Readmission | Carbapenem resistant *N* = 1711 | | | | | | Carbapenem susceptible *N* = 3129 | | | | | |
| --- | --- | --- | --- | --- | --- | --- | --- | --- | --- | --- | --- | --- |
|  | Overall  *N* = 1711 | Blood  *N* = 104 | Respiratory  *N* = 651 | Urine  *N* = 211 | Wound  *N* = 647 | Other  *N* = 98 | Overall  *N* = 3129 | Blood  *N* = 510 | Respiratory  *N* = 720 | Urine  *N* = 435 | Wound  *N* = 1236 | Other  N = 228 |
| Readmission due to any cause, n (%)^*^ | | | | | | | | | | | | |
| Overall | 999 (58.4) | 61 (58.7) | 345 (53.0) | 130 (61.6) | 398 (61.5) | 65 (66.3) | 1778 (56.8) | 298 (58.4) | 337 (46.8) | 293 (67.4) | 293 (67.4) | 472 (57.6) |
| Within 30 days | 432 (25.3) | 27 (26.0) | 166 (25.5) | 52 (24.6) | 164 (25.4) | 23 (23.5) | 703 (22.5) | 125 (24.5) | 158 (21.9) | 119 (27.4) | 85 (19.5) | 154 (18.8) |
| Days between discharge from index hospitalization to first readmission, median (Q1–Q3) | 41 (12.0–105.0) | 34 (14.0–82.0) | 34 (9.0–87.0) | 49  (12.0–122.0) | 44 (15.0–111.0) | 53 (15.0–118.0) | 51 (13.0–163.0) | 47 (12.0–137.0) | 36 (8.0–155.0) | 49 (16.0–153.0) | 39 (13.0–116.0) | 73 (19.5–23.0) |
| Readmission with any Gram-negative organism isolated, n (%)^*,†^ | | | | | | | | | | | | |
| Overall | 623 (36.4) | 33 (31.7) | 225 (34.6) | 91 (43.1) | 229 (35.4) | 45 (45.9) | 694 (22.2) | 86 (16.9) | 137 (19.0) | 141 (32.4) | 284 (23.0) | 46 (20.2) |
| Within 30 days | 197 (11.5) | 10 (9.6) | 79 (12.1) | 28 (13.3) | 71 (11.0) | 9 (9.2) | 176 (5.6) | 26 (5.1) | 44 (6.1) | 37 (8.5) | 54 (4.4) | 15 (6.6) |
| Days between discharge from index hospitalization to first readmission, median (Q1–Q3) | 58 (23.0–151.0) | 53 (27.0–113.0) | 51 (19.0–134.0) | 72 (25.0–211.0) | 59 (24.0–152.0) | 77 (37.0–173.0) | 94 (30.0–246.0) | 68.5 (23.0–194.0) | 60 (22.0–197.0) | 94 (30.0–255.0) | 117 (39.0–298.0) | 62 (19.0–112.0) |
| Readmission with *A. baumannii*, n (%)^*^. | | | | | | | | | | | | |
| Overall | 305 (17.8) | 14 (13.5) | 125 (19.2) | 39 (18.5) | 105 (16.2) | 22 (22.5) | 125 (4.0) | 15 (2.9) | 36 (5.0) | 24 (5.5) | 42 (3.4) | 8 (3.5) |
| Within 30 days | 106 (6.2) | 4 (3.9) | 47 (7.2) | 14 (6.6) | 37 (5.7) | 4 (4.1) | 41 (1.3) | 9 (1.8) | 11 (1.5) | 5 (1.2) | 11 (0.9) | 5 (2.2) |
| Days between discharge from index hospitalization to first readmission, median (Q1–Q3) | 51 (20.0–122.0) | 75 (27.0–133.0) | 46 (19.0–111.0) | 53 (16.0–258.0) | 57 (23.0–125.0) | 68 (41.0–113.0) | 79 (18.0–185.0) | 29 (12.0–100.0) | 71.5 (19.50–119.0) | 147 (52.0–284.0) | 105 (24.0–252.0) | 23 (8.0–43.5) |

^*^Unless otherwise specified.

^†^Most commonly isolated Gram-negative organisms included *Escherichia coli*, *Enterobacter cloacae*, *Klebsiella aerogenes*, *Klebsiella oxytoca*, *Klebsiella pneumoniae*, *Morganella morganii*, *Proteus mirabilis*, *Serratia marcescens*, *Acinetobacter baumannii*, *Pseudomonas aeruginosa*, *Stenotrophomonas maltophilia*.
