# Supplementary material for: Burden of illness in carbapenem-resistant Acinetobacter baumannii infections in US hospitals between 2014 and 2019
Source: BMC Infect Dis. 2022 Jan 6;22:36. doi: 10.1186/s12879-021-07024-4 (PMC8740340; doi:10.1186/s12879-021-07024-4)
Supplement: Supplementary file 7 — Additional file 7: Table S6. Algorithm to impute antibiotic susceptibility for Acinetobacter baumannii without susceptibility testing result for treatment given within 72 h of index culture. [file 12879_2021_7024_MOESM7_ESM.docx]

**Table S6.** Algorithm to impute antibiotic susceptibility for *Acinetobacter baumannii* without susceptibility testing result for treatment given within 72 hours of index culture

| Imputed **active** treatment algorithm | |
| --- | --- |
| Antibiotics received but not tested for susceptibility or missing interpretable results | Equivalent antibiotics from which susceptibility testing results can be used |
| doripenem | cefepime |
| imipenem | cefepime |
| meropenem | cefepime |
| cefepime | ceftazidime |
| doripenem | ceftazidime |
| imipenem | ceftazidime |
| meropenem | ceftazidime |
| levofloxacin | ciprofloxacin |
| polymyxin b | colistimethate na |
| imipenem | doripenem |
| meropenem | doripenem |
| doripenem | imipenem |
| meropenem | imipenem |
| ciprofloxacin | levofloxacin |
| doripenem | meropenem |
| imipenem | meropenem |
| colistimethate na | polymyxin b |
| doxycycline | tetracycline |
| minocycline | tetracycline |
| tigecycline | tetracycline |
